# Comparison of blood loss between intra-articular microporous polysaccharide hemospheres powder and tranexamic acid following primary total knee arthroplasty

**DOI:** 10.1038/s41598-024-55871-3

**Published:** 2024-03-02

**Authors:** Young-Dae Jeon, Jae-Ryong Cha, Jae-Min Oh, Sang-Gon Kim, Ki-Bong Park

**Affiliations:** Department of Orthopedic Surgery, Ulsan University Hospital, University of Ulsan College of Medicine, 25 Daehakbyeongwon-ro, Dong-gu, Ulsan, 44033 Republic of Korea

**Keywords:** Knee, Total knee arthroplasty, Blood loss, Hemostatic, Microporous polysaccharide hemospheres, Tranexamic acid, Health care, Medical research

## Abstract

Total knee arthroplasty (TKA) is associated with substantial blood loss and tranexamic acid (TXA) effectively reduces postoperative bleeding. Although it is known that there is no difference between intravenous or intra-articular (IA) injection, the general interest is directed towards topical hemostatic agents regarding thromboembolic events in high-risk patients. This study aimed to compare the blood conservation effects of IA MPH powder and TXA in patients undergoing primary TKA. We retrospectively analyzed 103 patients who underwent primary TKA between June 2020 and December 2021. MPH powder was applied to the IA space before capsule closure (MPH group, n = 51). TXA (3 g) was injected via the drain after wound closure (TXA group, n = 52). All patients underwent drain clamping for three postoperative hours. The primary outcome was the drain output, and the secondary outcomes were the postoperative hemoglobin (Hb) levels during the hospitalization period and the perioperative blood transfusion rates. An independent Student’s t-test was used to determine differences between the two groups. The drain output in the first 24 h after surgery was significantly higher in the MPH group than in the TXA group. The postoperative Hb levels were significantly lower in the MPH group than in the TXA group. In patients with simultaneous bilateral TKA, there was a significant difference in the blood transfusion volumes and the rates between groups. It is considered that IA MPH powder cannot replace IA TXA because of an inferior efficacy in reducing blood loss and maintaining postoperative Hb levels in the early postoperative period after primary TKA. Moreover, in the case of simultaneous bilateral TKA, we do not recommend the use of IA MPH powder because it was notably less effective in the field of transfusion volume and rate.

## Introduction

Total knee arthroplasty (TKA) is associated with substantial blood loss ranging from 1000 to 2000 mL^1^. Additionally, greater postoperative bleeding is known to increase pain, the risk of hematoma formation, wound infection, anemia, and the need for blood transfusions, all the while the range of motion of the knee joint decreases^2–4^.

Tranexamic acid (TXA), an inhibitor of fibrinolysis, effectively reduces postoperative bleeding. It is convenient in its application as there is no difference between administration methods, such as intravenous (IV) or intra-articular (IA) injection^5–8^. However, the preventive use of IV TXA regarding thromboembolic events in high-risk patients remains limited because of a lack of supporting evidence^9^, while the general interest is directed towards topical hemostatic agents^10,11^. Arista^®^ AH Absorbable Hemostatic Particles (Davol Inc., Warwick, RI, USA) is such a topical agent which harnesses the physical and chemical properties of hydrophilic microporous polysaccharide hemospheres (MPH) to assist in the control of blood loss^12^. In the field of TKA, there were two studies to evaluate MPH effectiveness for reduction of blood loss. Gleason et al.^13^ suggested application of MPH may be unnecessary and Yang et al.^14^ reported that MPH powder reduces blood loss in TKA patients compared to a placebo. However, to the best of our knowledge, no studies have compared the efficacy of intra-articularly applied MPH powder and TXA.

We hypothesized that the IA application of MPH powder would show an efficacy similar to that of TXA. To test this hypothesis, this study compared the effects of intra-articularly applied MPH powder and TXA on the blood conservation of patients who underwent primary TKA.

## Methods

### Study design

Clinical data was retrospectively collected from patients who underwent primary TKA at our institution between June 2010 and December 2021. Patients with degenerative osteoarthritis who underwent primary unilateral or simultaneous bilateral TKA were included in this study and were administrated an IA hemostatic agent during operation. After the time when MPH powder could be used in our institution, we used MPH powder for all patients undergoing TKA. Prior to using MPH powder, we used TXA for all patients. On the contrary, patients with rheumatoid arthritis, osteonecrosis, or post-traumatic arthritis were not eligible for this study. Exclusion criteria also included a history of coagulopathy, intake of antiplatelet or anticoagulant agents, and second surgery for staged bilateral TKA. Data from a total of 103 patients was analyzed. The intervention group, after this also referred to as MPH group, received 3 g of topical MPH in the IA space before capsule closure (n = 51), whereas the control group, also called TXA group, received 3 g of topical TXA in the IA space via a drain after wound closure (n = 52). This dose was chosen based on the findings of a double-blind randomized controlled trial that reported the application of a high dose of 3 g topical TXA was 43% more effective in reducing postoperative blood loss than a low dose of 500 mg in primary TKA^15^.

### Demographic data

The preoperative characteristics of the patients, including age, sex, body height, body weight, body mass index, preoperative hemoglobin (Hb), hematocrit, prothrombin time, and activated partial thromboplastin time, were collected.

### Surgical technique

All TKAs were performed by the same surgeon using the standard medial parapatellar approach under general anesthesia. A pneumatic tourniquet was inflated to a pressure of 300 mmHg before skin incision and deflated after the cement hardened. All TKAs were cemented using Triathlon posterior-stabilized implants (Stryker, Kalamazoo, MI, USA). Intramedullary guides were used for femoral cutting and extramedullary guides for tibial cutting. The entry point for the femoral intramedullary rod was closed using an autologous bone plug. The patella was not resurfaced in any of the cases. An IA drainage tube was placed in the knee joint and connected to a vacuum bag. The bag was not fully compressed for 3 h, followed by full compression until removal.

### Postoperative care protocols

All patients received an oral iron supplement (160 mg/day) for 4 weeks after surgery. To prevent deep vein thromboses, elastic stockings and intermittent pneumatic compression devices were used after surgery. After removal of suction drain, patients were administered antiplatelet agents or anticoagulants, the doses of which were the same as those prescribed preoperatively. Patients that had not been taking any antiplatelet agents or anticoagulants before surgery received 10 mg rivaroxaban (Xarelto®, Bayer Schering Pharma AG, Wuppertal, Germany), to be taken orally once daily for 14 days. The recovery process of all patients was accompanied by the same TKA rehabilitation protocols starting at postoperative day (POD) 1 and onwards. It focused on a continuous passive mobilization at their bedside and included active range of motion exercises, calf pump exercises, straight leg raises, and quadriceps strengthening exercises. The suction drain was removed at POD 3. All patients were allowed to walk with a knee brace to constraint the knee extension and a walker.

### Outcome assessment

The primary outcome measure of the study was drain output. The daily amount of postoperative drain output was monitored at the postoperative 0–24, 24–48 and 48–72 h. The secondary outcome measures included Hb levels during the hospitalization period and the rate of perioperative blood transfusions. We used the same critical pathway during the patient admission period and conducted blood tests on the same days before and after surgery. During the hospitalization period, postoperative Hb levels were checked on the day of surgery and on PODs 2, 3, 7, and 14. Hb levels were recorded in the outpatient clinic at postoperative week four. Changes in Hb levels were calculated and compared between the groups. The trigger for allogeneic transfusion of red blood cells was set at an Hb level of 7 g/dL in healthy patients who underwent unilateral TKA and between 8 and 9 g/dL in patients with clinical symptoms and signs of anemia. In patients undergoing simultaneous bilateral TKA, transfusion triggers included the above criteria, and a more than 3.0 g/dL decline of the postoperative Hb levels compared to those prior to the operation. The volume and rate of blood transfusion were recorded for all patients.

### Statistical analysis

An independent Student’s t-test was used to determine differences between the two groups in terms of demographic characteristics, preoperative clinical data, postoperative drainage amount, postoperative Hb levels, and blood transfusion rates. Furthermore, patients that underwent unilateral or simultaneous bilateral TKA each formed a subgroup of which the data were analyzed separately. Finally, we conducted additional analyses comparing the daily Hb levels of patients with and without blood transfusions between treatment groups. All statistical analyses were performed using the IBM SPSS Statistics for Windows (version 24, IBM Corp., Armonk, NY, USA). A *p* value less than or equal to 0.05 was considered to indicate statistical significance.

### Ethics approval

This study was approved by the Institutional Review Board (IRB) of Ulsan University Hospital (IRB No. UUH-2021-11-039-001) and was performed in line with the principles of the Declaration of Helsinki.

### Consent to participate

Informed consent was obtained from all individual participants included in the study.

## Results

The baseline demographics, including age, sex, body height, body weight, and body mass index, between the two groups (treatment with MPH or TXA) were not significantly different (Table 1). Analyses of the subgroups, unilateral or simultaneous bilateral TKA, did not show any significant differences, either. Furthermore, there were no significant differences in the preoperative serological test results.

**Table 1:** Comparison of the patient’s baseline demographics amongst treatment groups.

	Total population			Unilateral TKA			Bilateral TKA		
	MPH	TXA	*p* value	MPH	TXA	*p* value	MPH	TXA	*p* value
Number	51	52		40	40		11	12	
Female (%)	72.5	82.7	0.22	67.5	85.0	0.07	90.9	75.0	71.5
Age (years)	71.5	74.0	0.06	71.6	73.6	0.13	71.6	73.8	0.34
Height (cm)	157.1	155.0	0.18	157.6	154.8	0.09	155.3	153.8	0.63
Weight (kg)	66.6	66.4	0.94	67.2	66.1	0.67	64.6	64.0	0.89
BMI (kg/m^2^)	27.0	28.0	0.26	27.1	27.9	0.39	26.7	27.1	0.78
Hb (g/dL)	12.9	12.8	0.81	13.0	12.7	0.38	12.5	13.1	0.18
Hct (%)	38.9	38.7	0.72	39.2	38.4	0.35	37.9	39.7	11.2
PT (s)	11.2	11.7	0.14	11.1	11.9	0.11	11.4	11.2	0.68
aPTT (s)	30.0	30.2	0.69	39.8	30.3	0.47	30.6	29.9	0.45

*TKA* total knee arthroplasty, *MPH* microporous polysaccharide hemosphere, *TXA* tranexamic acid, *BMI* body mass index, *Hb* hemoglobin, *Hct* hematocrit, *PT* prothrombin time, *aPTT* activated partial thromboplastin time.

**Figure 1:**
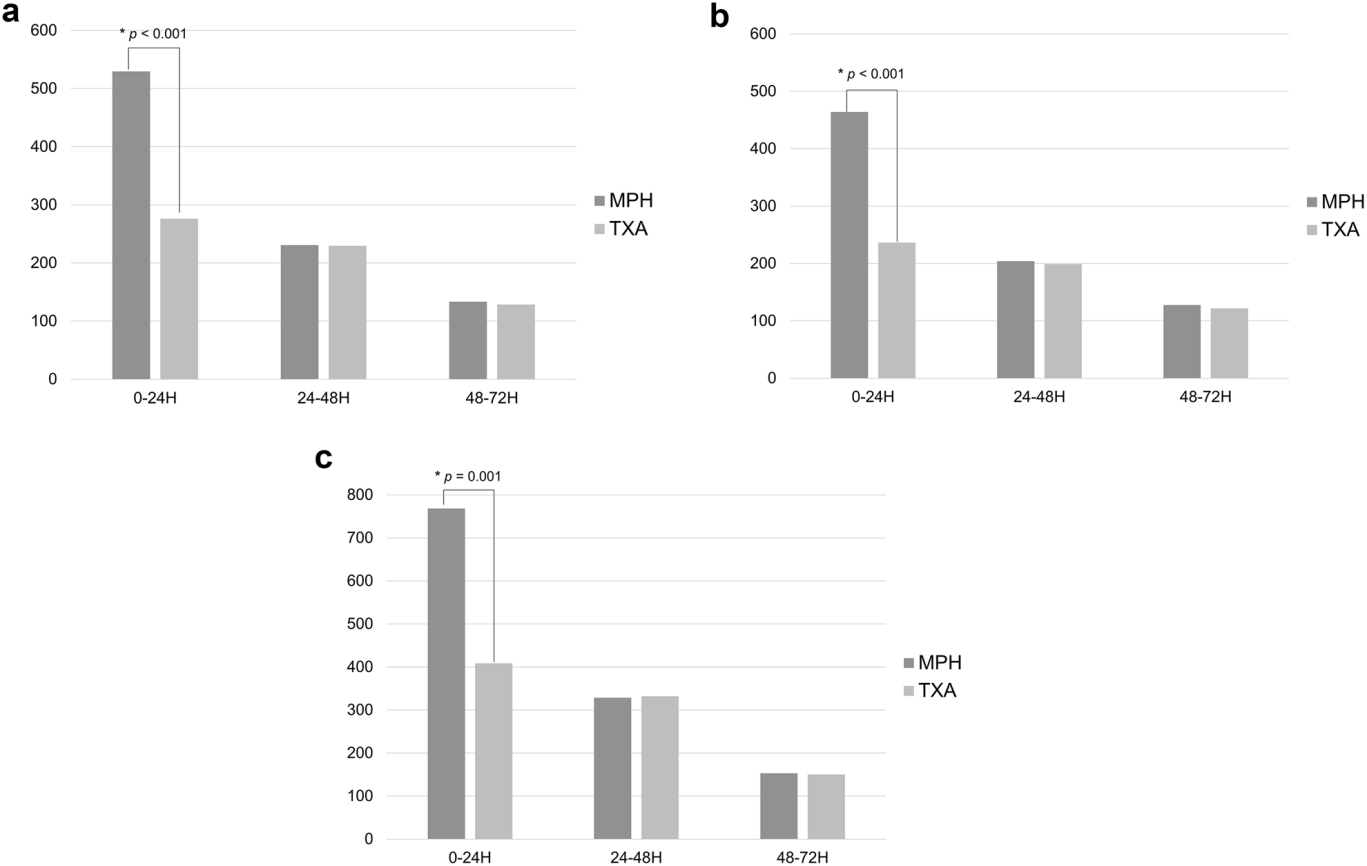
Postoperative amount of drain output between MPH powder and TXA. (**a**) All cases. (**b**) Unilateral total knee arthroplasty. (**c**) Bilateral total knee arthroplasty.

Regardless of being unilateral or simultaneous bilateral TKA, the amount of drain output in the first 24 h after surgery was significantly higher in the MPH group (529.7 mL) than in the TXA group (276.1 mL, p < 0.001) (Fig. 1A); however, there was no significant difference in the amount of drain output that was measured every 24 h thereafter. In patients who underwent unilateral TKA (Fig. 1B), the amount of drain output in the first 24 h after surgery was significantly higher in the MPH group (464.1 mL) than in the TXA group (236.4 mL, p < 0.001); however, there was no significant difference in the amount of drain output that was measured every 24 h thereafter. In the subgroup of patients with simultaneous bilateral TKA (Fig. 1C), MPH group also had a significantly greater drain output than the TXA group (768.3 mL and 408.6 mL, respectively, p = 0.001) in the first 24 h after surgery; however, there was no significant difference in the amount of drain output thereafter.

**Figure 2:**
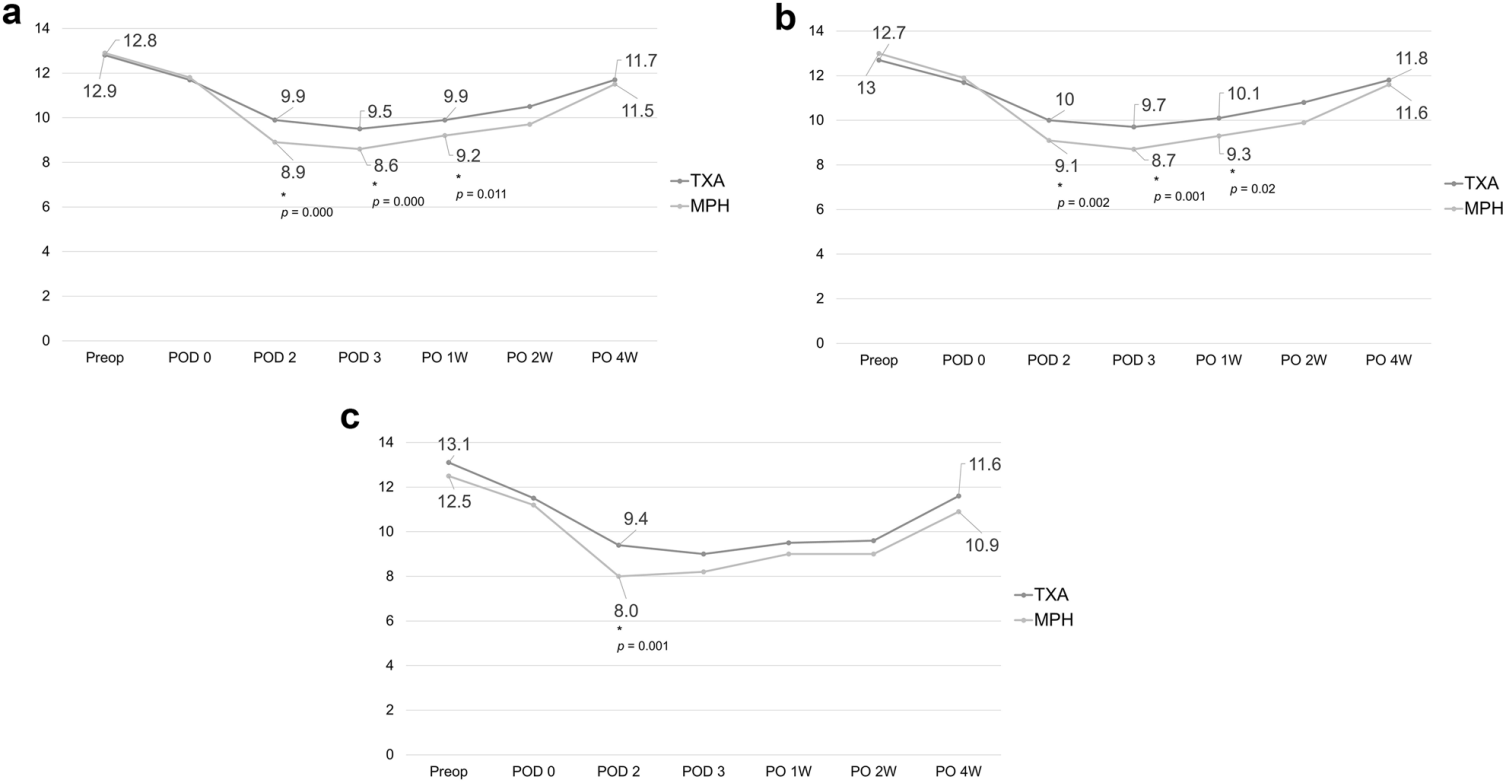
Changes in hemoglobin levels between MPH powder and TXA. (**a**) All cases. (**b**) Unilateral total knee arthroplasty. (**c**) Bilateral total knee arthroplasty.

Postoperative Hb levels were significantly lower on the second, third, and seventh day after surgery in the MPH group compared to those in the TXA group (Fig. 2A). Patients of both treatment groups who underwent unilateral TKA had the lowest Hb levels on the third day after surgery and then showed a tendency to recover gradually (Fig. 2B). Postoperative Hb levels were significantly lower on the second, third, and seventh day after surgery in the MPH group compared with those in the TXA group. Among patients who underwent simultaneous bilateral TKA (Fig. 2C), the MPH and TXA group had the lowest Hb levels on the postoperative day 2 and 3, respectively. Postoperative Hb levels were significantly lower on the second day after surgery in the MPH group compared with those in the TXA group.

Table 2 shows the changes in Hb levels among the treatment groups. The difference in Hb between preoperative and POD 3 and preoperative and POD 7 was significantly greater in the MPH group than in the TXA group. Among patients who underwent unilateral TKA, differences in Hb between preoperative and POD 3 and preoperative and POD 7 were significantly greater in the MPH group than in the TXA group. However, no significant difference was observed in the change in Hb before and after surgery between the two groups at any measurement point among patients who underwent simultaneous bilateral TKA.

**Table 2:** Comparison of the changes in hemoglobin levels among the treatment groups.

Hb (g/dL)	Total population			Unilateral TKA			Bilateral TKA		
	MPH	TXA	*p* value	MPH	TXA	*p* value	MPH	TXA	*p* value
Preop-POD3	5.0	3.3	< 0.001	4.3	3.1	< 0.001	4.3	4.1	0.76
Preop-PO1W	3.6	2.9	0.006	3.7	2.7	0.001	3.5	3.7	0.70
Preop-PO2W	3.2	2.3	0.10	3.1	2.7	0.19	3.6	4.4	0.43
Preop-PO4W	1.4	1.2	0.35	1.3	0.8	0.06	1.8	1.6	0.67

*Hb* hemoglobin, *TKA* total knee arthroplasty, *MPH* microporous polysaccharide hemosphere, *TXA* tranexamic acid, *POD* postoperative day, *PO* postoperative, *W* week.

The total transfusion volume was significantly higher in the MPH group (465.5 mL) than that in the TXA group (106.7 mL, p < 0.001). However, there were no significant differences in the total volume and transfusion rate in patients who underwent unilateral TKA. On the other hand, the transfusion rate in patients who underwent simultaneous bilateral TKA were significantly different between the MPH (100%) and TXA group (25.0%, p < 0.001).

## Discussion

The results of our retrospective trial show that the use of IA MPH powder during TKA led to similar postoperative drain output compared with the use of TXA. Nonetheless, patients of the MPH group lost significantly more blood in the first 24 h after surgery. These findings are congruent with a study conducted in the USA determined the efficacy of MPH powder in TKA patients and reported that more expected surgical blood loss and more postoperative blood loss occurred in the first 48 h after surgery^13^.

Generally, there are several options for reducing blood loss in TKA, such as the use of intraoperative induced-hypotensive anesthesia, tourniquet, patient-specific instrumentation, hemostatic agents, administration methods (systematic or topical), routes of local TXA administration, drain clamping, and clamping duration^16^. In this case our options to reduce blood loss were limited to tourniquets, IA hemostatic agents, and drain clamping for postoperative three hours. This is in line with findings of a systematic review and meta-analysis which reported TXA plus drain clamping as an efficient method for controlling blood loss after TKA^17^. However, the American Academy of Orthopaedic Surgeons published clinical practice guidelines and reported that strong evidence supports not using a drain in patients with TKA because there is no difference in the rate of complications or outcomes^18^.

These studies focused on the use of TXA as topical hemostatic agent and did not include MPH. We believe that our results may be related to the mechanism of action of MPH powder itself, rather than to other methods that reduce blood loss. Coagulation factors aggregate as they are absorbed by MPH, which forms a mechanical hemostatic plug that is effective for local areas. However, this might be ineffective in TKA surgery, during which diffuse bleeding occurs in large amounts^13^.

Regardless of whether MPH and TXA were used, and whether TKA was unilateral or bilateral, the decrease in Hb on the third postoperative day was greatest compared with preoperative Hb. The average decrease in Hb on POD 3 in the MPH group was 4.3 g/dL, regardless of whether unilateral or simultaneous bilateral TKA was performed. All patients who underwent simultaneous bilateral TKA using MPH powder required blood transfusion during hospitalization. We believe that the actual decrease in Hb would be more than 4.3 g/dL. There was a significant difference in Hb reduction between the two groups up to PO 1W (except for bilateral TKA), but there was no significant difference between the two groups at PO 2W and 4W. Therefore, it can be inferred that the capacity of MPH to prevent initial blood loss is lower than that of TXA.

Among patients who underwent unilateral TKA, when only those who did not need blood transfusions were analyzed separately, 94.6% of patients with IA TXA and 93.5% of patients with MPH powder recovered with daily Hb levels above 10 g/dL at postoperative week four. These results are superior to those of a previous study that investigated the natural course of Hb levels after unilateral TKA with IA TXA, where 89.7% of all patients recovered above the Hb level of 10 g/dL at postoperative week four^19^. Currently, staged bilateral TKA is conducted 1 or 2 weeks after the first surgery at many centers. At our institution, the anesthesiology department recommends that a patient’s Hb level should be at least 10 g/dL before TKA. To comply with this recommendation, the results of the previous study and this study suggest increasing the time frame between surgeries and to only perform the second surgery at least 4 weeks after the first one or after taking other measures that can increase the Hb level.

It is still controversial whether iron supplementation is necessary during TKA or whether it is better to alternate between oral and IV iron supplementation^20^. Among patients who underwent simultaneous bilateral TKA and IA TXA in this study, when only those who did not receive transfusion were analyzed separately, the Hb level at 4 weeks postoperatively recovered to 88.6% of the preoperative Hb level. Although there is a difference in that IV iron supplementation was administered to patients with staggered bilateral TKA, it has been reported that the Hb level at 4 weeks postoperatively in the group administered with IV iron and the group without IV iron recovered to 96.7% and 93.1% of the preoperative Hb level, respectively^21^. Although the design of this study using oral iron supplementation and that of a previous study using IV iron supplementation is not completely consistent, we considered IV iron supplementation is more helpful in Hb recovery at 4 weeks postoperatively.

Antifibrinolytic agents are known to reduce the transfusion rate in simultaneous bilateral TKA, and a previous study reported that the transfusion rates in the groups without and with antifibrinolytic agents were 75% and 30%, respectively^22^. In the simultaneous bilateral TKA group in this study, the rates of patients who received a transfusion in the MPH and TXA groups were 100% and 25%, respectively. Because the transfusion rate of the TXA group was lower than that reported in previous studies^22–24^, we considered that the degree of transfusion performed in those patients was not excessive. We believe that the reason for the high transfusion rate in the MPH group as follows: first, the indicators for transfusion appropriateness used in Korea suggest 7 g/dL of Hb level as a criterion for transfusion in unilateral TKA^25^, but there are no such criteria for simultaneous bilateral TKA. Second, regardless of unilateral or simultaneous bilateral TKA, patients who received MPH powder had a large amount of drain output (mean, 768.3 mL) accompanied by a great decline in Hb level (mean, 4.5 g/dL) in the first 24 h after surgery. We assume that the attending physicians preemptively chose to administer blood transfusions based on the general clinical progress of each patient. However, since this a retrospective study, we are not able to present the transfusion criteria for the simultaneous bilateral TKA patient group.

There are other limitations to this study, which include its retrospective design. We could not analyze the effects of oral iron supplements and antithrombotic drugs administered to all the patients. Furthermore, the clinical results related to bleeding were not presented, and safety evaluations, such as the occurrence of deep vein thrombosis, were not performed. Finally, the rate of perioperative transfusion was an indirect marker of blood loss and multifactorial. Rate of perioperative transfusion depends not only blood loss during and after surgery, but also on preoperative Hb levels, bleeding tendency, patient’s vascular anatomy, meticulous hemostasis, and operation time^26–28^. And patients with rheumatoid arthritis have higher probability of receiving blood transfusion postoperatively^29^.

## Conclusion

It is considered that IA MPH powder cannot replace IA TXA because of an inferior efficacy in reducing blood loss and maintaining postoperative Hb levels in the early postoperative period after primary TKA. Moreover, in the case of simultaneous bilateral TKA, we do not recommend the use of IA MPH powder because it was notably less effective in the field of transfusion volume and rate.

## Data Availability

The datasets used and analyzed during the current study available from the corresponding author on reasonable request.
